# Targeting cysteine-rich angiogenic inducer-61 by antibody immunotherapy suppresses growth and migration of non-small cell lung cancer

**DOI:** 10.3892/etm.2022.11365

**Published:** 2022-05-11

**Authors:** Xinpeng Li, Naxin Yuan, Lingdan Lin, Lixia Yin, Yiqing Qu

Exp Ther Med 16:730–738, 2018; DOI: 10.3892/etm.2018.6274

Following the publication of this paper, it was drawn to the attention of the Editorial Office by a concerned reader that the western blots featured in Fig. 4C and E on p. 736 appeared to have been misplaced, or were possibly mislabelled. After having asked the authors for an explanation to account for the problematic appearance of this figure, they realized that errors were made during the compilation of this figure, and supplied a new version of this figure to the office containing new data which rectifies the problems that were identified. The new version of Fig. 4 is shown below. All the authors agree with the publication of this corrigendum, and thank the Editor of *Experimental and Therapeutic Medicine* for allowing them the opportunity to publish this. Furthermore, they apologize to the readership for any inconvenience caused.

## Figures and Tables

**Figure 4:**
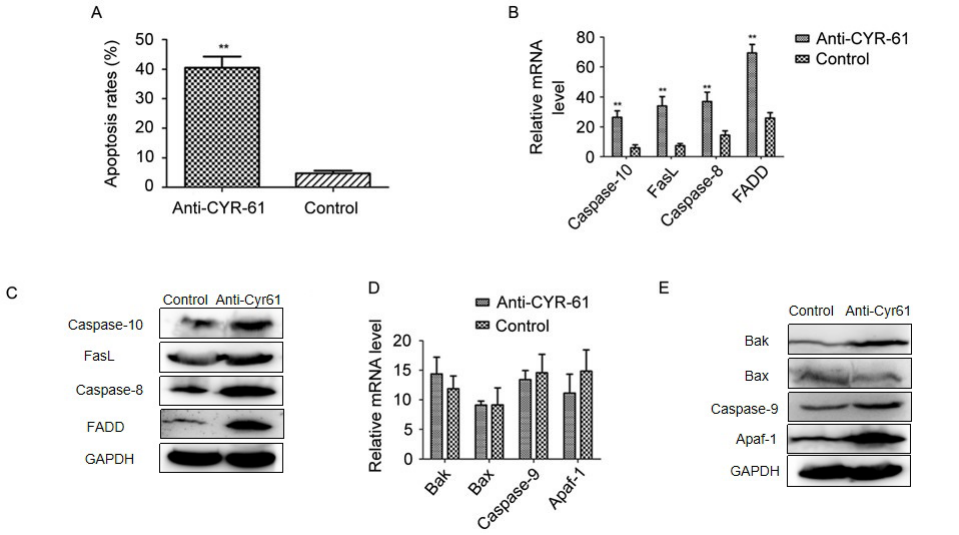
Effect of anti-CYR-61 on apoptosis in NSCLC cells. (A) Fluorescence-activated cell sorting was used to evaluate the apoptosis of H358 cells after anti-CYR-61 treatment. Apoptosis-related (B) mRNA and (C) protein expression levels in an exogenous cell apoptosis signaling pathway were analyzed in anti-CYR-61-treated cells. Apoptosis-related (D) mRNA and (E) protein expression levels in the mitochondrial apoptosis pathway were analyzed in anti-CYR-61-treated cells. Data are presented as the mean ± standard deviation of triplicate samples. ^**^P<0.01 vs. the control. CYR-61, cysteine-rich angiogenic inducer-61; NSCLC, non-small-cell lung cancer; FasL, Fas ligand; FADD, Fas-associated protein with Death Domain; Bak, B cell lymphoma-2 antagonist/killer; Bax, B cell lymphoma-2-associated X protein; Apaf-1, apoptotic protease activating factor 1.

